# Imipramine solubility-pH profiles: self-aggregation *vs.* common-ion effect

**DOI:** 10.5599/admet.3128

**Published:** 2025-12-31

**Authors:** Olivera S. Marković, Miloš P. Pešić, Alex Avdeef, Abu T. M. Serajuddin, Tatjana Ž. Verbić

**Affiliations:** 1 University of Belgrade - Institute of Chemistry, Technology and Metallurgy - National Institute of the Republic of Serbia, Department of Chemistry, Njegoševa 12, 11000 Belgrade, Republic of Serbia; 2 University of Belgrade - Faculty of Chemistry, Studentski trg 12-16, 11000 Belgrade, Republic of Serbia; 3 in-ADME Research, New York, NY 10128, USA; 4 St. John's University, College of Pharmacy and Health Sciences, 8000 Utopia Parkway, Queens, NY 11439, USA

**Keywords:** pH-ramp shake-flask method, solubility product, phosphate salt, chloride salt, critical micellar concentration, pH and buffer effect

## Abstract

**Background and purpose:**

The pH-dependent solubility of imipramine, a tricyclic antidepressant, and its hydrochloride salt was investigated in phosphate buffers and chloride-containing aqueous media using the pH-ramp shake-flask method. It was reported that aggregation of imipramine in acidic media and its partial degradation in alkaline media complicate the determination of its solubility. This was further investigated with modified methods.

**Experimental approach:**

For imipramine solubility studies, the computer program *p*DISOL-X was used to design experiments, process data, and refine the equilibrium constants. Isolated solid precipitates under various conditions were characterized using thermogravimetric analysis, differential scanning calorimetry, powder X-ray diffraction, and elemental analysis. The critical micelle concentration of imipramine hydrochloride was determined in 0.10 mol L^-1^ NaH_2_PO_4_ and in 0.15 mol L^-1^ NaCl by conductometric titrations.

**Key results:**

A detailed analysis of imipramine pH-solubility profiles reveals complex equilibria in the aqueous phase, as well as various solid-phase transformations. Intrinsic solubility of imipramine, solubility products of imipramine hydrochloride and imipramine phosphate salts, and aggregation constants (trimer, heptamer, and cationic complex with phosphate ions) were determined. Solid state characterization results are in accordance with pDISOL-X analysis.

**Conclusion:**

These findings, along with our previous solubility studies of desipramine and nortriptyline, suggest that even subtle structural variations can lead to significant differences in the aqueous media behaviour of tricyclic antidepressants. This type of information can be valuable in the early stages of drug discovery, in formulation optimization experiments, as well as in *in vitro* and *in vivo* studies.

## Introduction

**Figure 1.**
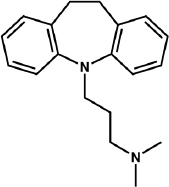
Structure of Imp

Imipramine (Imp, 3-(10,11-dihydro-5*H*-dibenzo[b,f]azepin-5-yl)-*N*,*N*-dimethylpropan-1-amine, Figure 1.) is a well-known tricyclic antidepressant (TCAs) drug that has gained much interest in the chemical and pharmaceutical literature because of its low aqueous solubility and surface-active properties [1]. It is a basic compound with a p*K*_a_ value of 9.52 (25 °C, *I* = 0.15 mol L^-1^). Its aqueous log molar intrinsic solubility (log *S*_0_) is -4.30 ± 0.26 (25 °C, average of 11 reported values), which is 14 μg mL^-1^ in the free base equivalent [2,3].

All TCAs are known to undergo self-aggregation, and there have been extensive studies on the critical micelle concentration (CMC) of imipramine using its hydrochloride salt (imipramine hydrochloride; ImpHCl). The results are summarized in Table 1 [4-8], which highlight how the CMC is dependent on applied experimental conditions like temperature, solvent used, ionic strength, pH, background electrolytes, etc. Although multiple values for Imp CMC have been published, no data are available about the CMC in phosphate-buffered solutions that are often recommended for use in pharmaceutical studies.

**Table 1.** Literature data on critical micelle concentration of imipramine HCl in aqueous media with experimental conditions and method of determination.

Method	Temperature (°C)	Additional solution information	CMC	Ref.
light scattering	30	N.A.^a^	50 mmol kg^-1^	[4]
conductivity	30	N.A.	48 mmol kg^-1^	[4]
pH method	30	N.A.	42 mmol kg^-1^	[4]
conductivity	25	N.A.	56.7 mmol L^-1^	[5]
surface tension	25	N.A.	38 mmol kg^-1^	[6]
surface tension	25	acetate buffer solution, pH 4	35 mmol kg^-1^	[6]
surface tension	25	50 mmol kg^-1^ NaCl	33 mmol kg^-1^	[6]
surface tension	27	0-400 mmol L^-1^ NaCl	47.5-21.6 mmol L^-1^	[7]
dye solubilisation	27	0-400 mmol L^-1^ NaCl	48.3-33.1 mmol L^-1^	[7]
surface tension	27	0.25-1.00 mmol L^-1^ CTAB^b^	37.8-3.2 mmol L^-1^	[7]
dye solubilisation	27	0.25-1.00 mmol L^-1^ CTAB	38.0-3.8 mmol L^-1^	[7]
surface tension	27	0.075-0.300 mmol L^-1^ TX-100^c^	33.3-4.3 mmol L^-1^	[7]
dye solubilisation	27	0.075-0.300 mmol L^-1^ TX-100	38.4-3.2 mmol L^-1^	[7]
conductivity	25	N.A.	39.8 mmol kg^-1^	[8]
conductivity	25	100 mmol kg^-1^ NaCl	35.8 mmol kg^-1^	[8]
conductivity	25	300 mmol kg^-1^ urea	44.0 mmol kg^-1^	[8]

^a^Not available

^b^cetyltrimethylammonium bromide

^c^polyethylene glycol t-octylphenyl ether.

In our previous research, we investigated the pH-dependent solubility of desipramine (Ds) and nortriptyline (Nor), two other TCAs, in phosphate and/or chloride-containing heterogeneous systems [9-11]. Both compounds formed cationic and anionic complexes with phosphates, resulting in increased drug solubility. The influence of suspension composition, direction of pH change in titrations (low-to-high or high-to-low), pH, and buffer effect on the aqueous solubility of drugs was explored. Additionally, multiple solid phase transformations during the determination of solubility *vs*. pH were characterized (hydrochloride salt, phosphate salts, and free base).

As part of our ongoing study of poorly soluble TCAs, this paper considers the solubility of imipramine as a function of pH in the presence of multiple counterions [9-11]. Specifically, the solubility of imipramine was investigated by the pH-Ramp shake-flask method (pH-RSF method) in the presence of chloride and/or phosphate counterions. Solubility data were analysed using the computer program *p*DISOL-X^TM^ (*in*-*ADME* Research) [12-14]. Different solid phases formed in equilibria with solutions were analysed using thermogravimetric analysis (TGA), differential scanning calorimetry (DSC), powder X-ray diffraction (PXRD), and elemental analyses. Critical micelle concentration of ImpHCl at 25.0 °C was determined by conductometric titrations.

Since measurements were done by the same research group and similar experimental conditions were applied, it is possible to compare all TCA solubility results and draw conclusions about important factors affecting TCA solubility. It is demonstrated that specific structural characteristics can influence the aqueous solution chemistry of TCAs. This type of information can be valuable in the early stages of drug discovery, in formulation optimization experiments, as well as in *in vitro* and *in vivo* studies. Furthermore, our papers in the Ds, Nor, and Imp trilogy emphasize the importance of experimental design in drug solubility research.

## Experimental

### Chemicals and reagents

Imipramine hydrochloride (≥99 % TLC purity) was purchased from Sigma-Aldrich. All other chemicals, like sodium dihydrogen phosphate dihydrate, disodium hydrogen phosphate dihydrate, hydrochloric acid, sodium hydroxide, acetic acid, and phosphoric acid, were purchased from multiple reputable vendors, and they were of analytical quality or better. Millipore-purified water was used for the preparation of all aqueous solutions.

### pH measurement and conversion to the pH scale

The Crison pH-Burette 24 2S (Crison Instruments, Alella, Barcelona, Spain) equipped with the Hach 52 09 micro-combination pH electrode (Hach, Loveland, Colorado, USA) was used to measure pH values. The electrode was calibrated by standard Hach buffer solutions (pH 4.01, 7.00 and 9.21).

Since the reported equilibrium constants are based on the concentration scale, *i.e.*, the ‘constant ionic medium’ thermodynamic standard state [1], the operational pH-meter values were converted to those based on the concentration scale, pH (= -log *C*_H⁺_), using the Avdeef-Butcher equation [15]:



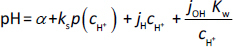

(1)


where α corresponds to the negative logarithm of the activity coefficient of H^+^ at the working temperature and ionic strength; the *k*_s_ term denotes the ratio between the actual slope and the Nernst slope; *K*_w_ is the ionization constant of water, taken as a function of temperature and ionic strength [16]. The *j*_H_ term corrects pH readings for any nonlinearity due to liquid junction and glass asymmetry potentials in highly acidic solutions (pH < 1.5), whereas the *j*_OH_ term corrects for high pH (pH > 11.5) nonideal behaviour.

### Solubility determination using the pH-RSF method

Six different procedures were applied to determine pH-dependent solubility of Imp in suspensions containing phosphate plus chloride ions (Sets 1, 2, and 4), solely chloride ions (Sets 3 and 5; phosphate-free titrations), and solely phosphate ions (Set 6; chloride-free titrations). All measurements were performed at 25 ± 1°C. The equilibration time was 24 hours (6 hours of stirring + 18 hours of sedimentation) and the phases were separated by centrifugation. Details of stock solution preparation, titration, and solubility data are given in the Supplementary material (Tables S1 to S6).

Sets 1 and 2 (phosphate + chloride containing suspensions; low-to-high titrations): Acidified stock solutions (pH 1.90 to 2.09) were prepared by mixing 7.50 to 9.50 mL of 0.15 mol L^-1^ NaH_2_PO_4_, 0.500 mL of 0.973 mol L^-1^ HCl, and 0.5464 to 0.5650 g of ImpHCl_(s)_. Aliquots of acidic stock solution (1.000 mL) were placed into vials and initial pH values were measured. Standardized 0.908 mol L^-1^ NaOH was used for sample pH adjustment within the pH range 3.82 to 11.43 (measured after 24 hours of equilibration). All solids in the suspensions dissolved at pH <3.82.

Set 3 (phosphate-free suspensions; low-to-high titration): Samples were prepared by adding 0.15 mol L^-1^ NaCl in vials containing weighed ImpHCl_(s)_ and initial pH values were measured. Then, standardized 0.908 mol L^-1^ NaOH was used for pH adjustment within pH range 7.37 to 11.87 (measured after 24 hours of equilibration). All solids in the suspensions dissolved at pH <7.37.

Set 4 (phosphate + chloride containing suspensions; high-to-low titration): Stock suspension (pH 6.13) was prepared by adding 4.50 mL of 3.00 mol L^-1^ NaH_2_PO_4_ and 3.50 mL of 1.062 mol L^-1^ NaOH in the vial containing 0.5287 g Imp (oil). The stock suspension was divided into vials (1.000 mL aliquots) and the pH of each sample was adjusted using 1.073 mol L^-1^ HCl within the pH range 2.67 to 6.27 (pH measured after 24 hours of equilibration).

Set 5 (phosphate-free suspensions; high-to-low titration): stock suspension (pH 7.93) was prepared by mixing 9.00 mL of 1.70 mol L^-1^ NaCl and 0.6374 g Imp (oil). Aliquots of stock suspension (1.000 mL) were placed into vials and pH was adjusted using standardized 1.073 mol L^-1^ HCl within the pH range 2.62-8.00 (measured after 24 hours of equilibration).

Set 6 (chloride-free suspensions; low-to-high titration): stock suspension (pH 2.24) was prepared by adding 7.00 mL of 2.00 mol L^-1^ NaH_2_PO_4_ and 3.00 mL of 2.00 mol L^-1^ H_3_PO_4_ in the vial containing 0.6914 g Imp (oil). Stock suspension was divided into vials (1.000 mL aliquots). After initial pH measurements, pH values were adjusted using standardized 1.062 mol L^-1^ NaOH within the pH range 2.50 to 6.37 (measured after 24 hours of equilibration).

Imp (oil) used in Sets 4 to 6 was prepared by mixing 3.00 mL of 1.062 mol L^-1^ NaOH and 0.7067 to 0.8424 g ImpHCl_(s)_ (pH 11.53 to 12.75). After centrifugation, the oil was washed 3 times with 200 μL of Millipore-purified water.

### HPLC analysis for imipramine concentration

Imp concentrations in the supernatant solutions during the solubility determination were analysed by Agilent Technologies HPLC 1260 Infinity Series instrument with DAD detector (Santa Clara, CA, USA) and HPLC LC2050C 3D PDA (Shimadzu, Japan). Chromatographic separation was conducted using a Hypersil Gold 50×3 mm column packed with 5 μm particles at a flow rate of 0.5 mL min^-1^, a detection wavelength of 252 nm, and a column temperature of 25 °C. The mobile phases used for the gradient elution were the mixtures of 1 % acetic acid in water (A) and acetonitrile (B) in the following steps: (a) from 70 % A + 30 % B to 100 % B during 5 min, (b) 100 % B for 1 min, and (c) back to 70 % A + 30 % B during 1 min. Post run: 5 min.

### Conductometric titrations

Conductometric titrations were performed using Conductivity Pocket Meter Cond 330i (WTW, Germany) equipped with TetraCon 325 (WTW, Germany) conductometric cell (cell constant 0.48 cm^-1^) calibrated with N.I.S.T. standard KCl solution 1413 μS cm^-1^ (25 °C). Accurately weighed portions of ImpHCl were added to 8.00 mL of 0.15 mol L^-1^ NaCl or 0.10 mol L^-1^ NaH_2_PO_4_ solutions at 25.0 ± 0.1 °C.

### Solid state characterization: sample preparation

Samples of solid precipitates that represent different Imp suspensions during the determination of solubility were isolated for solid-state characterization, where Sample 1 was isolated from Imp suspensions containing phosphate ions (chloride-free suspensions), Samples 2 to 5 were isolated from Imp suspensions containing phosphate and chloride ions, and Sample 6 was isolated from Imp suspensions containing chloride ions (phosphate-free suspension). The equilibration time was 24 hours (6 hours of stirring followed by 18 hours of sedimentation) for all samples, and the phases were separated by centrifugation. The solids were dried in a vacuum chamber at room temperature for 3 days prior to analysis. The detailed procedures for the preparation of the samples for the solid-state characterization are given below:

Sample 1 (pH 1.26) was obtained by adding 0.500 mL of 2.00 mol L^-1^ NaH_2_PO_4_ and 2.90 mL of 2.00 mol L^-1^ H_3_PO_4_ in a vial containing 0.1807 g Imp (oil).

Sample 2 (pH 2.67) was prepared by adding 1.000 mL of 2.00 mol L^-1^ NaH_2_PO_4_ and 0.700 mL of 1.073 mol L^-1^ HCl in a vial containing 0.1971 g of Imp (oil).

Sample 3 (pH 6.21) was prepared by mixing 1.80 mL of 0.15 mol L^-1^ Na_2_HPO_4_, 0.05 mL of 1.073 mol L^-1^ HCl and 0.2367 g of ImpHCl_(s)_.

Sample 4 (pH 8.28) was obtained by adding 1.30 mL of 0.15 mol L^-1^ Na_2_HPO_4_ and 0.200 mL of 1.062 mol L^-1^ NaOH in a vial containing 0.2478 g ImpHCl_(s)_.

Sample 5 (pH 12.55) was obtained by mixing 1.00 mL of 0.15 mol L^-1^ Na_2_HPO_4_, 1.00 mL of 1.062 mol L^-1^ NaOH and 0.2590 g of ImpHCl_(s)_.

Sample 6 (pH 12.87) was prepared by mixing 0.2741 g of ImpHCl_(s)_, 0.70 mL of 0.15 mol L^-1^ NaCl and 1.30 mL of 1.0621 mol L^-1^ NaOH.

Imp (oil) used for preparation of Samples 1 and 2 was prepared by mixing 0.8639 g of ImpHCl_(s)_ and 3.00 mL of 1.062 mol L^-1^ NaOH (pH 12.89). After centrifugation, the isolated Imp (oil) was washed twice with HPLC-grade water.

### Elemental analysis

The elemental analysis of the samples from Set 1 was performed using a Vario EL III C,H,N,S/O Elemental Analyser (Elementar Analysensysteme GmbH, Hanau-Germany) *via* combustion analysis. The samples from Set 1 were air-dried for 3 days before the analysis.

### Thermogravimetric analysis

The TGA Q50 thermogravimetric analyser (TA Instruments, DE, USA) was used to determine the weight loss as a function of temperature. The samples (5.54 to 12.00 mg) were heated from approximately 25 to 400 °C at a heating rate of 10 °C min^-1^ under a nitrogen atmosphere.

### Differential scanning calorimetric analysis

The thermal behaviour of solids was scanned as a function of temperature using a Q200 differential scanning calorimeter (TA Instruments, DE, USA). Each sample (8.6 to 9.8 mg) was sealed in a Tzero pan with a pinhole and heated to 250 °C at a rate of 5 °C min^-1^, with a modulation of ±1.0 °C per minute. The results were analysed using the Universal Analysis software version 2000 (TA Instruments).

### Powder X-ray diffraction analysis

The powder X-ray diffractometer (Shimadzu 6000, Kyoto, Japan) was used to obtain PXRD patterns at 25 ± 1 °C with a monochromatic CuKα radiation source operated at 40 kV and 30 mA. The scanning rate of 2 ° min^-1^ was used over the 2*θ* range of 10 to 60°. The test materials were placed as thin layers inside glass sample holders.

## Results and discussion

### Solubility analysis

**Figure 2.**
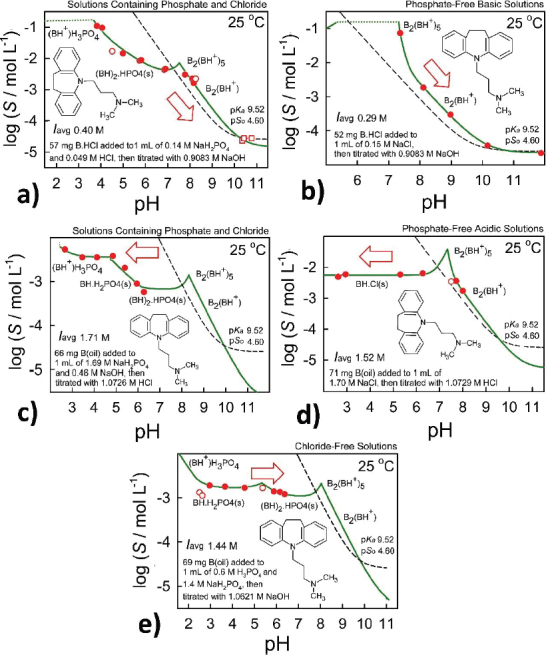
Solubility-pH profiles for Imp at 25 °C. (a) Sets 1 and 2 (Imp samples containing chloride and phosphate). (b) Set 3 (phosphate-free Imp samples). (c) Set 4 (Imp samples containing chloride and phosphate). (d) Set 5 (phosphate-free Imp samples). (e) Set 6 (chloride-free Imp samples). Dashed curves were calculated with the Henderson-Hasselbalch equation. The dotted lines represent pH regions where the solutions were subsaturated. The unfilled circles denote outlier points assigned zero weights in the refinement. The unfilled squares denote points where degradation was observed. In the drawings, the symbol ‘B’ represents the imipramine free base.

The results of the determination of pH *vs.* solubility of Imp by the pH-RSF method, as described in Sets 1 to 6 under the Experimental section, are shown graphically in sub-figures a to e of Figure 2, where the solid curves represent calculated Imp solubility profiles and the dashed curves were calculated with the Henderson-Hasselbalch (HH) equation. The detailed titration and solubility data used to generate the curves are presented in the Supplementary material (Tables S1 to S6).

The computer program *p*DISOL-X was used to simulate solubility-pH profiles, guide experimental design, process the data, and refine the equilibrium constants. All equilibrium constants were scaled to *I*_ref_ = 0.15 mol L^-1^ (reference ionic strength). The applications of the program and the mathematical approach (including automatic ionic strength compensation) used in *p*DISOL-X log *S*-pH simulation-refinement were described previously [12-14]. Refined constants are shown in Table 2.

**Table 2.** Summary of *p*DISOL-X calculations^a^

Set	log *K*_141_ (BH^+^) H_3_PO_4_	SD	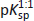 (BH)H_2_PO_4_	SD	p*K*_sp_^2:1^ (BH)_2_HPO_4_	SD	*l*og *K*_310_ B_2_(BH^+^)	SD	log *K*_750_ B_2_(BH^+^)_5_	SD	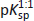 BHCl	SD	p*S*_0_ B
1,2^b^	3.039	0.94	-	-	6.699	0.06	8.735	0.06	8.165	0.94	-	-	4.602
3^c^	-	-	-	-	-	-	8.816	0.06	9.981	0.06	2.338	fixed	4.602
4^d^	0.364	0.58	2.672	0.05	7.045	0.04	8.816	fixed	9.981	fixed	-	-	4.602
5^e^	-	-	-	-	-	-	8.332	0.13	9.816	0.47	2.338	0.02	4.602
6^f^	0.324	0.13	2.829	0.01	6.801	0.01	8.816	fixed	9.981	fixed	-	-	4.602
wt mean	0.374	0.016	2.823	0.00010	6.812	0.00009	8.728	0.0017	9.970	0.0040	2.338		4.602
sd	1.56		0.11		0.18		0.26		1.00		0.02		0.02
Set	*I*_avg_ (M)				*n*				GOF				
1,2^b^	0.395				17				1.30				
3^c^	0.288				5				0.54				
4^d^	1.709				7				1.40				
5^e^	1.518				7				0.65				
6^f^	1.440				9				0.29				

^a^Goodness-of-fit based on assigned standard deviation, SD = 0.05 log for each point. wt mean is calculated with SD values as weights. log*K* with triple index subscript refers to stoichiometric coefficients: B, H, PO_4_

^b^Chloride and phosphate suspensions (57 mg BHCl in 1 mL of 0.14 mol L^-1^ NaH_2_PO_4_ + 0.049 mol L^-1^ HCl; decomp. pH > 10)

^c^Chloride suspensions (52 mg BHCl in 1 mL of 0.15 mol L^-1^ NaCl; B(oily) pH > 8)

^d^Chloride and phosphate suspensions (68 mg B(oil) in 1 mL of 1.69 mol L^-1^ NaH_2_PO_4_ + 0.46 mol L^-1^ NaOH)

^e^Chloride suspensions (71 mg B(oil) in 1 mL of 1.70 mol L^-1^ NaCl)

^f^Phosphate suspensions (69 mg B(oil) in 1 mL of 0.6 mol L^-1^ H_3_PO_4_ + 1.4 mol L^-1^ NaH_2_PO_4_)

Sets 1 and 2 were prepared as imipramine suspensions containing phosphate and chloride ions *via* low-to- high titrations. These sets were analysed as one combined set (Figure 2a, Table 2). Equilibrium analysis suggested that the precipitates are imipramine hydrogen phosphate at a pH range of 3.82 to 7.85 (Equation 2) and imipramine free base at pH >7.85. Elevated solubility in the pH range 7 to 10 can be rationalized by the formation of cationic trimer and heptamer of imipramine (Equations 3 and 4). Furthermore, there was no precipitate formation at pH <3.82. High solubility near pH 4 is consistent with the formation of a cationic complex (Equation 5). The cationic complex ‘competes’ with the formation of imipramine salt at the acidic pH region. Cationic aggregate formation of various bases has previously been described in the literature [17-19], and the relevant aggregation constants are given below:



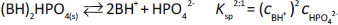

(2)




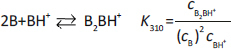

(3)




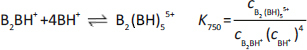

(4)




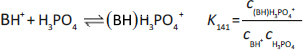

(5)


Set 3 (phosphate-free) was designed for intrinsic solubility determination of Imp (Figure 2b, Table 2). In this set, there was no precipitate formation below pH 7.37. Equilibrium analysis suggested that the solid phase at pH ≥ 7.37 was the Imp base. The data analysis suggested that water-soluble cationic imipramine trimer and heptamer (Equations (3) and (4)) were also formed at pH <10. This indicates a higher aggregation tendency of Imp in chloride-containing suspensions compared to chloride + phosphate-containing suspensions. The aggregation constants are higher in Set 3 than in Sets 1 and 2 (Table 2).

The addition of a much higher amount of ImpHCl (*c* = 1.12 mol L^-1^ at pH 1.9) in phosphate-containing solution (Sets 1 and 2) only produced clear but very viscous solutions, and, therefore, the accurate determination of solubility was not possible. To add missing data points to acidic region of log*S vs.* pH diagrams, Sets 4 to 6, were designed, where the mass-to-volume ratio remains similar as in Sets 1 to 3, but two experimental modifications were applied: first, Imp base was used as a starting material instead of ImpHCl, and, second, the counterions (phosphate and/or chloride) concentration was increased, whereby the average ionic strength, *I*_avg_, changed from 0.29 to 0.39, to *I*_avg_ 1.44 to 1.71 mol L^-1^.

It has been reported in the literature that self-aggregation of similar compounds decreases when the starting material is a base instead of a salt [2,17,20,21]. Furthermore, it is expected that the solubility of a drug would decrease when the counterions concentration is increased [22-25]. These modifications led to the successful determination of Imp saturation solubility in acidic media containing phosphate and/or chloride ions.

Set 4 (Figure 2c) was prepared as phosphate plus chloride containing Imp (base) suspensions with a raised total phosphate ion concentration (1.69 mol L^-1^ in initial stock suspension) compared to Sets 1 and 2 (0.14 mol L^-1^ in initial stock suspension). Equilibrium analysis showed that three precipitates were formed: imipramine dihydrogen phosphate (pH <5, Equation 6), imipramine hydrogen phosphate (pH 5 to 8, Equation 2) and Imp base (pH >8.5).



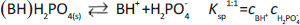

(6)


Set 5 (phosphate-free) was designed for ImpHCl solubility product determination (Figure 2d, Table 2), where the total chloride ion concentration was increased by using 1.70 mol L^-1^ NaCl as background electrolyte, instead of 0.15 mol L^-1^ NaCl (used in Set 3). The ImpHCl precipitate was isolated at pH_max_ <7.5 (Equation 7), and the Imp base was isolated at pH_max_ >7.5.



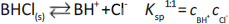

(7)


Set 6 (chloride-free) was designed for the determination of solubility products of imipramine dihydrogen phosphate and imipramine hydrogen phosphate salts (Figure 2e, Table 2). Total phosphate concentration in the initial stock suspension was 2.00 mol L^-1^. It is shown by equilibrium analysis that three precipitates were formed: imipramine dihydrogen phosphate (pH_max_ <5.5), imipramine hydrogen phosphate (5.5 < pH < 8), and imipramine base (pH_max_ >8).

These results (Figure 2) show that saturated solutions of Imp in the acidic region can be prepared under high ionic strength experimental conditions, when the total concentration of chloride or phosphate ions is increased as Imp base is used as the starting material instead of ImpHCl. In Sets 4 to 6, the mass-to-volume ratio was kept similar as in Sets 1 to 3. However, special attention must be paid to all potential problems that arise when ionic strength is high, as described previously in the white paper on solubility [26]. In this respect, automatic ionic strength compensation and the adjustment of pH electrode parameters from Avdeef-Bucher four-parameter equation [1,15] when the ionic strength is varied are very useful possibilities of the computer program *p*DISOL-X. Furthermore, the differences between solubility-pH profiles (solid green curves) and theoretical profiles calculated with the HH equation for pH >9 (Figure 2) are due to activity corrections made in the *p*DISOL-X calculations, which have been previously described in detail [14]. The value of p*S*_0_ = 4.60 is harmonized to the selected reference ionic strength, *I*_ref_ = 0.15 mol L^-1^, in accordance with the ‘constant ionic medium’ thermodynamic state [1] adopted in the calculations. The differences between the solid green curves and the Henderson-Hasselbalch curve for pH >9 in Figure 2 (Sets 4 to 6) are due to activity corrections using the Stokes-Robinson hydration theory, modified with the imipramine salting-out constant, *K*_s_ = 0.42 [14]. Sets 4 to 6 develop relatively high average ionic strengths in the alkaline solutions, ranging from 1.44 to 1.71 mol L^-1^. In contrast, Set 3 shows alignment in the alkaline region, since the ionic strength, *I* = 0.29 mol L^-1^, is much closer to the reference value, *I*_ref_ = 0.15 mol L^-1^.

### Conductometric titrations

Self-aggregation of Imp was observed during pH-dependent solubility experiments. As previously mentioned, multiple data about Imp CMC were published so far [4-8], but, to the best of our knowledge, there is no data about CMC in phosphate-buffered solutions, often recommended for use in pharmaceutical studies.

CMC values of ImpHCl were determined by conductometric titrations at 25.0 ± 0.1 °C. Titrations were designed to be as similar as possible to experimental conditions used in Sets 1 to 3 (pH regions without solid precipitate). Two CMC values for ImpHCl, 35.5±8.7 mmol L^-1^ in 0.10 mol L^-1^ NaH_2_PO_4_ and 26.2±2.3 mmol L^-1^ in 0.15 mol L^-1^ NaCl, were determined as the intersection point in the titration curves (Supplementary material, Figure S1).

Sodium chloride solution (0.15 mol L^-1^) was used for Imp suspensions preparation in Set 3 (phosphate-free), thus the same solution was used for CMC determination of ImpHCl in phosphate-free solution. Although a 0.15 mol L^-1^ NaH_2_PO_4_ solution was used in Sets 1 and 2 (chloride plus phosphate containing suspensions), it was not possible to determine the CMC value in this solution, as precipitation started before the CMC value was reached. This was the reason for using 0.10 mol L^-1^ NaH_2_PO_4_ solution for CMC determination of ImpHCl. This agrees with ‘competition’ between precipitation of imipramine phosphate salts and formation of Imp aggregates in Sets 1 and 2 proposed according to solubility analysis (discussed in section Solubility analysis).

Solubility of Imp in pH range 3.82 to 4.06 in chloride plus phosphate containing suspensions (Sets 1 and 2) and in phosphate-free suspensions at pH 7.37 (Set 3) is above CMC values, which is in accordance with the Imp complex formation and the Imp self-aggregate formation (based on *p*DISOL-X analysis).

The trimer and heptamer formations were not observed in our previous studies of the pH-dependent solubility of desipramine and nortriptyline in chloride- and/or phosphate-containing suspensions [9-11]. Instead, cationic phosphate complex formation occurred at pH <4 in desipramine suspensions containing chloride and phosphate ions and at pH <2 in nortriptyline suspensions containing phosphate ions. An anionic phosphate complex is formed in the chloride plus phosphate-containing alkaline media. Both desipramine and nortriptyline have a secondary amino group at the end of the hydrocarbon chain and imipramine has a tertiary amino group. This structural difference can be the reason for the different composition of studied suspensions of imipramine compared to desipramine and nortriptyline suspensions.

### Imipramine solid-state characterization

Solid precipitates isolated from Set 1 were analysed by elemental analysis. The results, as presented in Table 3, are in good agreement with predictions from *p*DISOL-X calculations, and suggest that solid precipitates are hydrated imipramine hydrogen phosphate salt in the pH range 4.06 to 6.85 and hydrated imipramine base in the alkaline media.

**Table 3.** Elemental analysis of solid precipitates isolated from Imp Set 1 suspensions.

pH	C content, %	H content, %	Proposed compound composition
4.06	63.64	8.17	(ImpH)_2_HPO_4_×4H_2_O
4.99	61.06	7.79	(ImpH)_2_HPO_4_×4H_2_O
5.81	65.21	7.65	(ImpH)_2_HPO_4_×2H_2_O
6.85	64.04	7.67	(ImpH)_2_HPO_4_×2H_2_O
8.09	66.79	8.30	Imp×4H_2_O^a^
8.11	66.57	8.06	Imp×4H_2_O^a^
8.15	71.55	8.14	Imp×2H_2_O

^a^pH value is near pH_max_, so solid precipitate might be a mixture of phosphate salt and Imp base

To perform TGA, DSC, and PXRD analyses, an additional six samples were prepared from chloride-free suspension (Sample 1), chloride + phosphate-containing suspensions (Samples 2 to 5), and phosphate-free suspension (Sample 6). To decrease the Imp solubility in the acid region, Samples 1 (pH 1.26) and 2 (pH 2.67) were prepared according to Sets 6 (chloride-free suspensions; higher phosphate concentration) and 4 (chloride ++ phosphate-containing suspension; higher phosphate concentration) experimental design, respectively. Samples 3 (pH 6.21), 4 (pH 8.28), and 5 (pH 12.55) were prepared according to the experimental design used in Sets 1 and 2 (chloride + phosphate containing suspension), and Sample 6 (pH 12.87) according to Set 3 design (phosphate-free suspensions).

**Figure 3.**
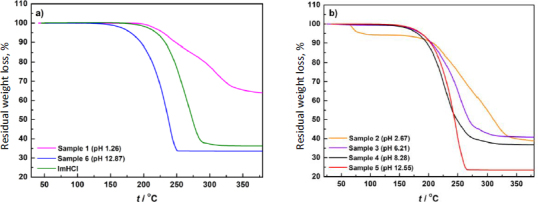
TGA scans of samples (residual weight loss *vs.* temperature): (a) 1 (isolated from chloride-free suspension), 6 (isolated from phosphate-free suspensions), and imipramine hydrochloride as reference. (b) 2 to 5 (isolated from suspensions containing phosphate and chloride ions)

According to the thermogravimetric analysis presented in Figure 3, all samples are anhydrous, except Sample 2 (pH 2.67), which is a hydrate having a weight loss of 5.8 % up to 100 °C.

**Figure 4.**
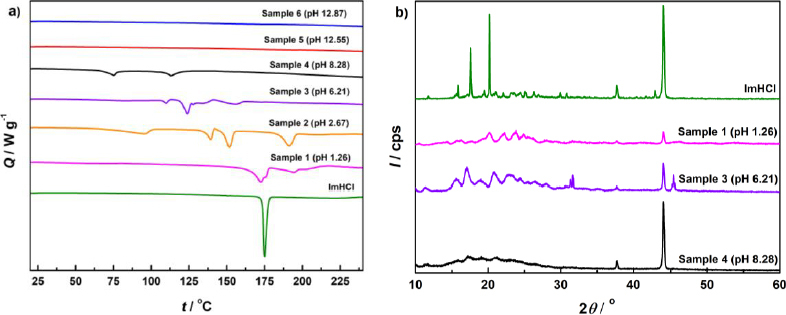
(a) 1 DSC scans (heat flow (*Q*) vs. temperature (*t*)) of Samples 1 to 6, and ImpHCl as reference. (b) PXRD patterns (diffraction intensity (*I*) *vs.* diffraction angle (2*θ*)) of ImpHCl as reference, and Samples 1, 3 and 4

The results of DSC and PXRD analyses of ImpHCl salt, as well as various samples prepared in this investigation, are presented in Figure 4a and Figure 4b, respectively. The reference ImpHCl salt exhibits a sharp melting endotherm at 175 °C (Figure 4a), and its PXRD pattern reveals a high degree of crystallinity (Figure 4b). The other samples analysed exhibit DSC scans and PXRD patterns different then ImpHCl salt ‘reference’, and their solid-state characteristics are described below.

DSC scans of Samples 5 and 6 (Figure 4a), isolated at pH 12.55 and pH 12.87, respectively, are featureless and without characteristic transitions, and they also do not exhibit any PXRD peaks (PXRD scans not shown), confirming that the isolated solids are an amorphous Imp base. These results demonstrate that an amorphous (oily) imipramine base was formed at alkaline pH conditions, which did not convert to any crystalline solid in 24 hours of equilibration.

*p*DISOL-X analysis shows that Samples 1 (pH 1.26) and 2 (pH 2.67) are consistent with the formation of imipramine dihydrogen phosphate. Sample 1 was prepared from phosphate-containing imipramine suspension (chloride-free) and Sample 2 from phosphate + chloride-containing imipramine suspension with increased phosphate concentration (total concentrations of phosphates and chlorides were 1.18 mol L^-1^ and 0.44 mol L^-1^, respectively). Therefore, it is expected that the phosphate salt will precipitate. Both the DSC scan and PXRD pattern show that Sample 1 is a crystalline solid. Sample 2 is imipramine dihydrogen phosphate monohydrate, according to the weight loss of 5.8 % obtained from TGA analysis.

Based on pH value of the solid phase transition between imipramine dihydrogen phosphate and imipramine hydrogen phosphate (pH_max_), it is expected that Sample 3 is imipramine hydrogen phosphate, since it was isolated at pH 6.21 (above pH_max_). The PXRD pattern of Sample 3 is different compared to the PXRD pattern of Sample 1 (pH 1.26), so Sample 3 is probably crystalline imipramine hydrogen phosphate, but it might also contain a small amount of imipramine dihydrogen phosphate.

Sample 4 (pH 8.28) was isolated at a pH value close to the range of solid phase transition from imipramine hydrogen phosphate to imipramine base. An amorphous halo is observed in the PXRD pattern of Sample 4. Hence, this sample could be a mixture of crystalline imipramine hydrogen phosphate salt and free base.

### Degradation of Imp in alkaline suspensions

It has previously been reported that there could be degradation of imipramine under certain pH conditions [27], which could influence the solubility data during the determination of solubility *vs.* pH. Therefore, the equilibrated solutions in the present investigation were analysed by HPLC for the presence of any degradation products. The HPLC analysis of solutions from Sets 1 and 2 during the determination of solubility at pH <10 and of samples from Set 3 at pH < 9 did not demonstrate any degradation of Imp, since only one peak was observed (Figure S2, Supplementary material). However, multiple peaks appeared in the chromatograms at pH >10 in samples from Sets 1 and 2 and at pH > 9 in samples from Set 3 (Figures S4 and S5, Supplementary material), implying partial Imp degradation.

It is noteworthy that partial degradation was observed by HPLC only in the saturated Imp solution when the solid phase was present in the system (experiments for solubility determination). Degradation was not observed after 24 hours of stirring at 25 °C in any of the Imp unsaturated solutions containing phosphates and/or chlorides (data was not shown). Since an equilibrium exists between the solution phase and the oily solid phase under alkaline pH conditions, it appears that degradation occurs in both phases. However, since, in the present investigation, the Imp concentrations in equilibrated solutions were determined by HPLC analysis, the solubility data at alkaline conditions were not compromised by degradation and reflected exact values.

## Conclusions

Detailed investigation of aqueous solubility-pH behaviour of tricyclic antidepressant Imp using pH-Ramp shake-flask method is presented in this study. High solubility of Imp in phosphate and chloride-containing suspensions at pH <4 and in phosphate-free suspensions at pH <7 prevented the preparation of saturated solutions. Solubility in these pH ranges is above the CMC value of ImpHCl determined by conductometric titrations. It is shown that Imp solubility can be diminished if the total concentration of counterion (phosphate or chloride) is increased or if Imp base is used instead of salt. It is observed that phosphate or chloride Imp salts precipitate in the acidic region, depending on the total counterion concentrations. Partial degradation of imipramine occurred at pH >10 in phosphate and chloride-containing suspensions and at pH >8.5 in chloride-containing suspensions (phosphate-free). This study emphasizes the importance of experimental design in drug solubility research and provides information that can be valuable in the early stages of drug discovery, in formulation optimization experiments, as well as in *in vitro* and *in vivo* studies.

## Supplementary material

Additional data are available at https://pub.iapchem.org/ojs/index.php/admet/article/view/3128, or from the corresponding authors on request.


