# Supplementary material for: Imipramine solubility-pH profiles: self-aggregation vs. common-ion effect
Source: ADMET DMPK. 2025 Dec 31;14:3128. doi: 10.5599/admet.3128 (PMC12994603; doi:10.5599/admet.3128)
Supplement: Supplementary file 1 [file ADMET-14-3128-S1.pdf]

ADMET & DMPK 00(0) (2025) S3128

Open Access : ISSN : 1848-7718

https://www.pub.iapchem.org/ojs/index.php/admet

Supplementary material to

Imipramine solubility-pH profiles: self-aggregation *vs.* common-ion effect

Olivera S. Marković[1], Miloš P. Pešić[2], Alex Avdeef[3], Abu T. M. Serajuddin[4] and Tatjana Ž. Verbić[2]

[1]*University of Belgrade - Institute of Chemistry, Technology and Metallurgy - National Institute of the Republic of Serbia, Department of Chemistry, Njegoševa 12, 11000 Belgrade, Republic of Serbia*
[2]*University of Belgrade - Faculty of Chemistry, Studentski trg 12-16, 11000 Belgrade, Republic of Serbia*
[3]*in-ADME Research, New York, NY 10128, USA*
[4]*St. John's University, College of Pharmacy and Health Sciences, 8000 Utopia Parkway, Queens, NY 11439, USA*

ADMET & DMPK **14** (2026) 3128; https://doi.org/10.5599/admet.3128

Table S1. Set 1, imipramine hydrochloride titration and solubility data

Vial	$pH_{initial}$	V_{NaOH} / µL[a]	pH_{final}	*S* / mmol L^{-1}	log(*S* / mol L^{-1})
1[b]	2.11	70.0	4.06	95.0	-1.02
2	2.12	100.0	4.99	14.4	-1.84
3	2.27	140.0	5.81	8.77	-2.06
4	2.13	180.0	6.85	4.29	-2.37
5	2.12	250.0	8.09	2.23	-2.65
6	2.10	260.0	8.11	2.21	-2.66
7	2.10	320.0	8.15	1.68	-2.78
8	2.10	350.0	8.17	1.61	-2.79
9[c]	2.12	450.0	10.77	0.0275	-4.56
10[c]	2.10	500.0	11.43	0.0276	-4.56

[a]c_{NaOH}=0.9083 mol L^{-1}; [b]Solubility above CAC of ImpHCl (CAC = 35.5 mmol L^{-1} in 0.10 mol L^{-1} NaH_2PO_4 at 25.0 ± 0.1°C. pH 4.45 to 4.33); [c]Degradation products present in supernatant

Table S2. Set 2, imipramine hydrochloride titration and solubility data

Vial	$pH_{initial}$	V_{NaOH} / µL[a]	V_{HCl} / µL[a]	pH_{final}	*S* / mmol L^{-1}	log(*S* / mol L^{-1})
1[c]	1.87	90.0	–	3.82	109	-0.96
2	1.82	132.0	–	4.51	16.9	-1.77
3	1.83	150.0	–	5.74	8.39	-2.08
4	1.81	200.0	5.0	6.90	4.62	-2.34
5	1.83	230.0	–	7.78	3.01	-2.52
6	1.84	290.0	–	8.26	2.21	-2.66
7	1.87	469.0	–	8.87	0.285	-3.54
8[d]	1.84	450.0	–	10.35	0.0227	-4.64

[a]c_{NaOH} = 0.9083 mol L^{-1}; [b]c_{HCl} = 0.9725 mol L^{-1}; [c]Solubility above CAC of ImpHCl (CAC = 35.5 mmol L^{-1} in 0.10 mol L^{-1} NaH_2PO_4 at 25.0 ± 0.1°C. pH 4.45 to 4.33); [d]Degradation products present in supernatant.

Table S3. Set 3, phosphate-free imipramine hydrochloride titration and solubility data

vial	m_{ImpHCl} / g	V_{NaCl} / mL[a]	$pH_{initial}$	V_{NaOH} /µL[b]	V_{HCl} / µL[c]	pH_{final}	*S* / mmol L^{-1}	log(*S* / mol L^{-1})
1[d]	0.05055	1.000	5.49	100.0	–	7.37	73.4	-1.13
2	0.05720	1.000	6.14	190.0	–	8.11	1.85	-2.71
3[e]	0.05010	1.000	6.34	174.0	3.0	8.98	0.297	-3.53
4[e]	0.05215	1.000	6.15	225.0	37.0	10.16	0.0367	-4.44
5[e]	0.05010	1.000	6.40	200.0	–	11.87	0.0222	-4.65

[a]0.15 mol L^{-1} NaCl; [b]c_{NaOH} = 0.9083 mol L^{-1}; [c]c_{HCl} = 0.9725 mol L^{-1}; [d]Solubility above CAC of ImpHCl (CAC=26.2 mmol L^{-1} in 0.15 mol L^{-1} NaCl at 25.0 ± 0.1°C. pH=5.92 – 5.17); [e]Degradation products present in supernatant.

Table S4. Set 4, imipramine titration and solubility data

Vial	$pH_{initial}$	V_{HCl} / µL[a]	pH_{final}	*S* / mmol L^{-1}	log(*S* / mol L^{-1})
1	6.33	700.0	2.67	5.35	-2.27
2	6.24	600.0	3.46	3.52	-2.45
3	6.29	570.0	4.13	3.56	-2.45
4	6.18	500.0	4.85	3.77	-2.42
5	6.30	300.0	5.40	2.02	-2.69
6	6.25	100.0	5.95	0.897	-3.05
7	6.28	–	6.27	0.580	-3.24

[a] c_{HCl} = 1.0729 mol L^{-1}

Table S5. Set 5, phosphate-free imipramine titration and solubility data

Vial	$pH_{initial}$	V_{HCl} / µL[a]	V_{NaOH} / µL[b]	pH_{final}	*S* / mmol L^{-1}	log(*S* / mol L^{-1})
1	8.00	125.0	55.0	2.62	4.89	-2.31
2	7.98	120.0	10.0	2.94	5.85	-2.23
3	8.07	115.0	40.0	5.30	5.78	-2.24
4	8.16	135.0	7.0	6.27	6.31	-2.20
5	7.91	110.0	–	7.50	3.43	-2.46
6	7.96	135.0	–	7.71	3.59	-2.45
7	7.93	–	–	8.00	1.71	-2.77

[a] c_{HCl} = 1.0729 mol L^{-1}; [b] c_{NaOH} = 1.0621 mol L^{-1}

Table S6. Set 6, chloride-free imipramine titration and solubility data

vial	$pH_{initial}$	V_{NaOH} / µL[a]	pH_{final}	*S* / mmol L^{-1}	log(*S* / mol L^{-1})
1	2.27	–	2.50	1.33	-2.88
2	2.32	60.0	2.63	1.11	-2.95
3	2.29	200.0	2.96	1.90	-2.72
4	2.28	300.0	3.66	1.79	-2.75
5	2.35	350.0	4.55	1.69	-2.77
6	2.31	500.0	5.37	1.67	-2.78
7	2.36	800.0	5.88	1.39	-2.86
8	2.36	700.0	6.16	1.35	-2.87
9	2.46	1000.0	6.37	1.21	-2.92

[a] c_{NaOH}=1.0621 mol L^{-1}

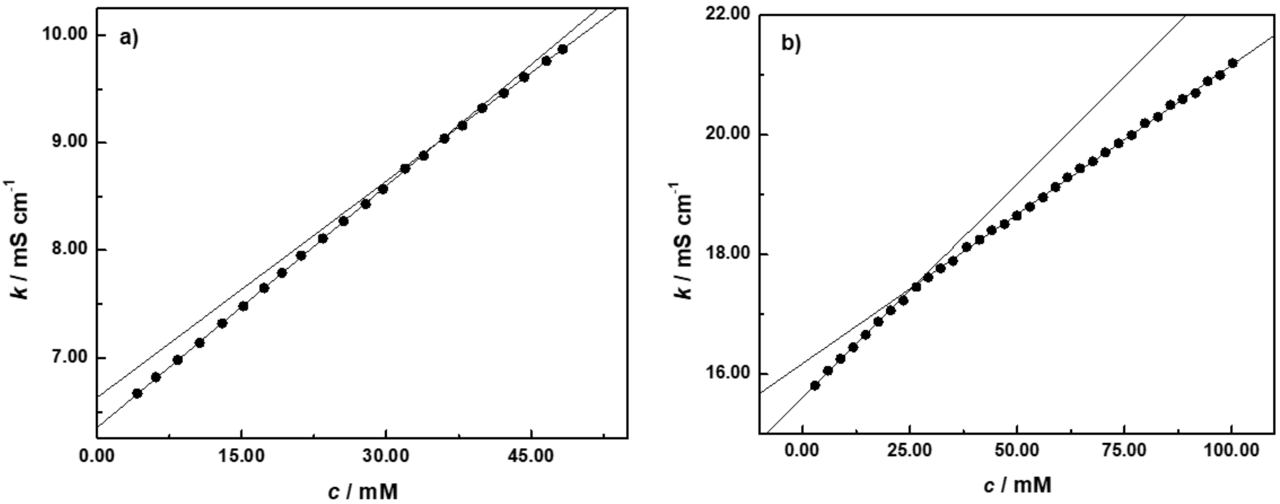

Figure S1. Conductometric titration curves of ImpHCl at 25.0 ± 0.1 °C in. (a) 0.10 mol L^{-1} NaH_2PO_4 (pH was 4.45 at the beginning of titration and 4.33 in the end of titration) and (b) 0.15 mol L^{-1} NaCl (pH was 5.92 at the beginning of titration and 5.17 in the end of titration)

CC BY

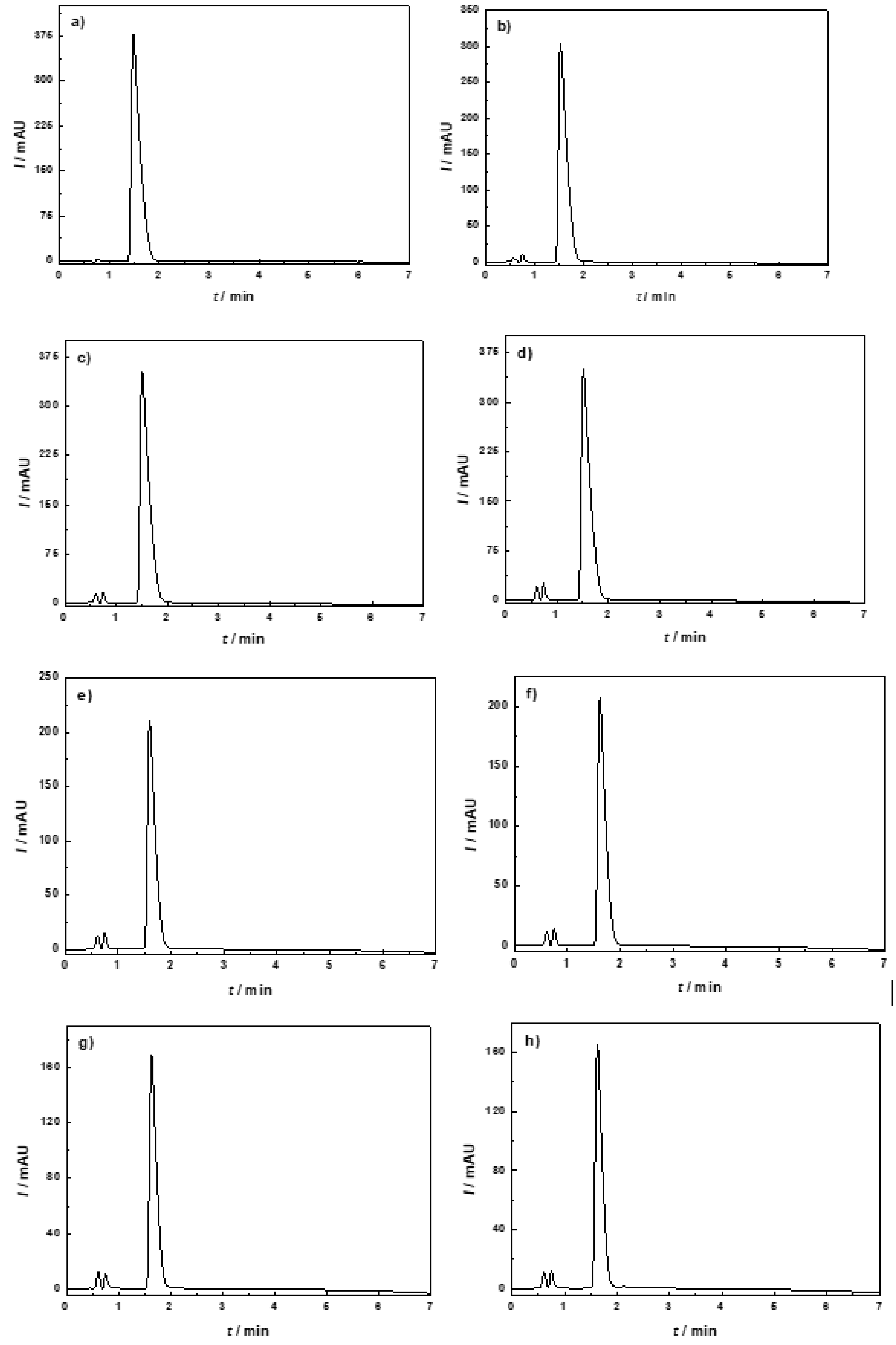

Figure S2. HPLC Chromatograms for Set 1 samples 1 to 8 at different pH values: (a) Sample 1 (pH 4.06, diluted 200×), (b) Sample 2 (pH 4.99, diluted 40×), (c) Sample 3 (pH 5.81, diluted 20×), (d) Sample 4 (pH 6.85, diluted 10×), (e) Sample 5 (pH 8.09, diluted 10×), (f) Sample 6 (pH 8.11, diluted 10×), (g) Sample 7 (pH 8.15, diluted 10×), (h) Sample 8 (pH 8.17, diluted 10×)

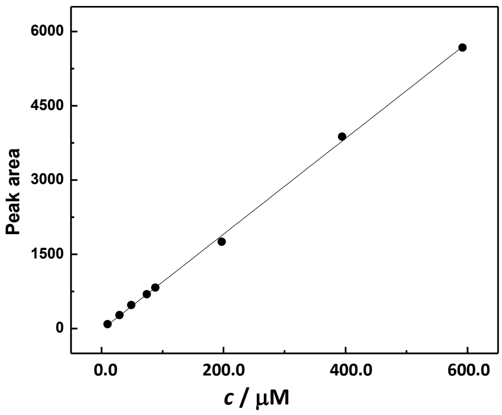

Figure S3. Calibration diagram for imipramine (concentration range 9.89 to 592 μmol L^{-1}), linear fit: peak area = $9.6655\times10^{6}c$ -25.9765; r^2=0.9989

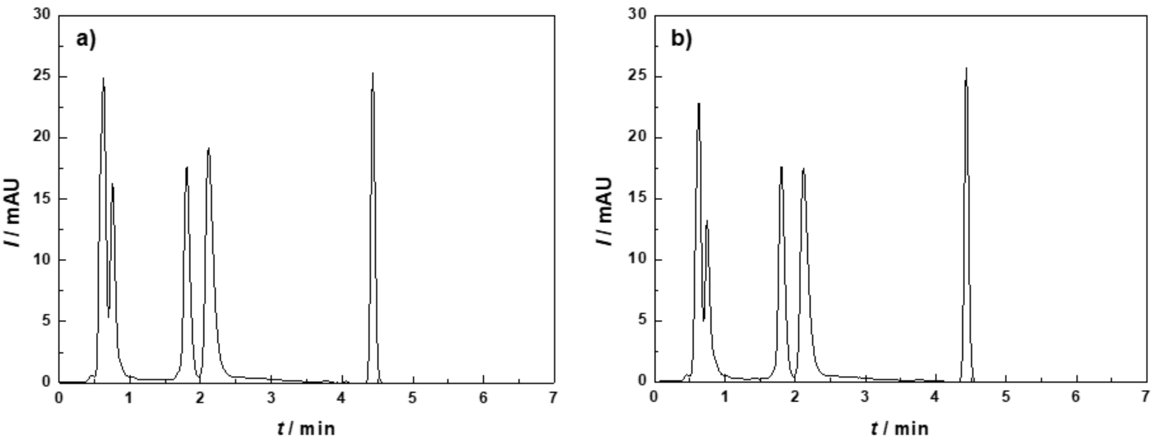

Figure S4. HPLC Chromatograms for Set 1 samples at different pH values (degradation products present): (a) Sample 9 (pH 10.77, diluted 2×), (b) Sample 10 (pH 11.43, diluted 2×). Detection wavelength: 252 nm

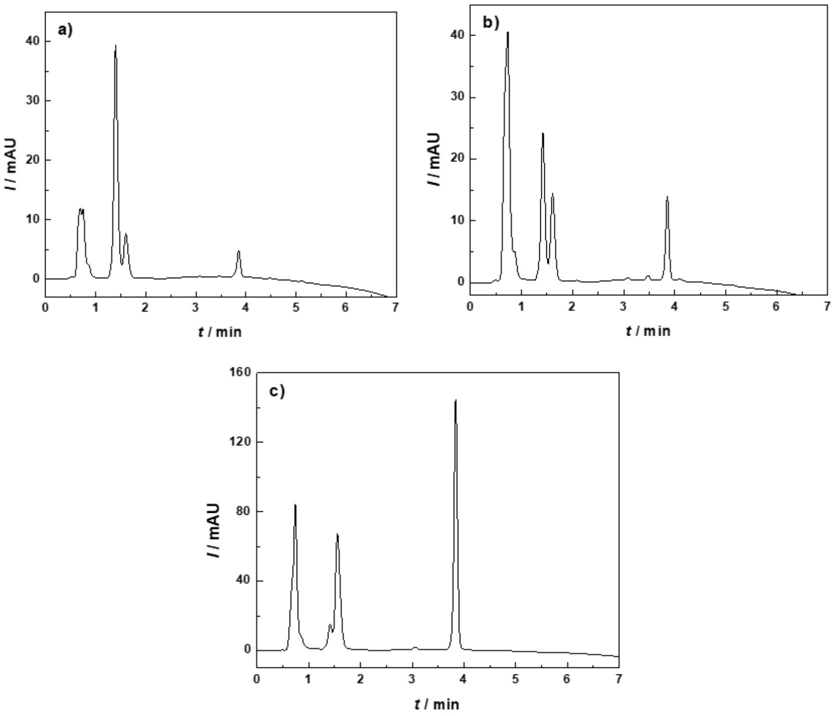

Figure S5. HPLC Chromatograms for Set 3 samples at different pH values (degradation products present): (a) Sample 4 (pH 8.98, diluted 10×), (b) Sample 5 (pH 10.16, diluted 2×), (c) Sample 6 (pH 11.87, diluted 2×). Detection wavelength: 252 nm

(cc) BY
