# Supplementary material for: Imipramine solubility-pH profiles: self-aggregation vs. common-ion effect
Source: ADMET DMPK. 2025 Dec 31;14:3128. doi: 10.5599/admet.3128 (PMC12994603; doi:10.5599/admet.3128)
Supplement: Supplementary file 2 [file ADMET-14-3128-S1.docx]

ADMET & DMPK 00(0) (2025) S3128

*
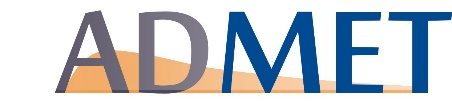
***Open Access : ISSN : 1848-7718**[***https://www.pub.iapchem.org/ojs/index.php/admet***](https://www.pub.iapchem.org/ojs/index.php/admet)

Supplementary material to

Imipramine solubility-pH profiles: self-aggregation *vs.* common-ion effect

Olivera S. Marković^1^ [
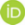
](https://orcid.org/0000-0001-5830-1445), Miloš P. Pešić^2^ [
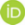
](https://orcid.org/0000-0002-1982-040X), Alex Avdeef^3^ [
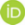
](https://orcid.org/0000-0002-3139-5442), Abu T. M. Serajuddin^4^ [
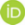
](https://orcid.org/0000-0002-2596-715X) and Tatjana Ž. Verbić^2^ [
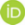
](https://orcid.org/0000-0002-6348-1644)

^1^University of Belgrade - Institute of Chemistry, Technology and Metallurgy - National Institute of the Republic of Serbia, Department of Chemistry, Njegoševa 12, 11000 Belgrade, Republic of Serbia

^2^University of Belgrade - Faculty of Chemistry, Studentski trg 12-16, 11000 Belgrade, Republic of Serbia

^3^in-ADME Research, New York, NY 10128, USA

^4^St. John's University, College of Pharmacy and Health Sciences, 8000 Utopia Parkway, Queens, NY 11439, USA

ADMET & DMPK **14** (2026) 3128; <https://doi.org/10.5599/admet.3128>

**Table S1.** Set 1, imipramine hydrochloride titration and solubility data

| Vial | pH_initial_ | *V*_NaOH_ / µL^a^ | pH_final_ | *S* / mmol L^-1^ | log(*S* / mol L^-1^) |
| --- | --- | --- | --- | --- | --- |
| 1^b^ | 2.11 | 70.0 | 4.06 | 95.0 | -1.02 |
| 2 | 2.12 | 100.0 | 4.99 | 14.4 | -1.84 |
| 3 | 2.27 | 140.0 | 5.81 | 8.77 | -2.06 |
| 4 | 2.13 | 180.0 | 6.85 | 4.29 | -2.37 |
| 5 | 2.12 | 250.0 | 8.09 | 2.23 | -2.65 |
| 6 | 2.10 | 260.0 | 8.11 | 2.21 | -2.66 |
| 7 | 2.10 | 320.0 | 8.15 | 1.68 | -2.78 |
| 8 | 2.10 | 350.0 | 8.17 | 1.61 | -2.79 |
| 9^c^ | 2.12 | 450.0 | 10.77 | 0.0275 | -4.56 |
| 10^c^ | 2.10 | 500.0 | 11.43 | 0.0276 | -4.56 |

^a^*c*_NaOH_=0.9083 mol L^-1^; ^b^Solubility above CAC of ImpHCl (CAC = 35.5 mmol L^-1^ in 0.10 mol L^-1^ NaH_2_PO_4_ at 25.0 ± 0.1**°**C. pH 4.45 to 4.33); ^c^Degradation products present in supernatant

**Table S2.** Set 2, imipramine hydrochloride titration and solubility data

| Vial | pH_initial_ | *V*_NaOH_ / µL^a^ | *V*_HCl_ / µL^a^ | pH_final_ | *S* / mmol L^-1^ | log(*S* / mol L^-1^) |
| --- | --- | --- | --- | --- | --- | --- |
| 1^c^ | 1.87 | 90.0 | – | 3.82 | 109 | -0.96 |
| 2 | 1.82 | 132.0 | – | 4.51 | 16.9 | -1.77 |
| 3 | 1.83 | 150.0 | – | 5.74 | 8.39 | -2.08 |
| 4 | 1.81 | 200.0 | 5.0 | 6.90 | 4.62 | -2.34 |
| 5 | 1.83 | 230.0 | – | 7.78 | 3.01 | -2.52 |
| 6 | 1.84 | 290.0 | – | 8.26 | 2.21 | -2.66 |
| 7 | 1.87 | 469.0 | – | 8.87 | 0.285 | -3.54 |
| 8^d^ | 1.84 | 450.0 | – | 10.35 | 0.0227 | -4.64 |

^a^*c*_NaOH_ = 0.9083 mol L^-1^; ^b^*c*_HCl_ = 0.9725 mol L^-1^; ^c^Solubility above CAC of ImpHCl (CAC = 35.5 mmol L^-1^ in 0.10 mol L^-1^ NaH_2_PO_4_ at
25.0 ± 0.1**°**C. pH 4.45 to 4.33); ^d^Degradation products present in supernatant.

**Table S3.** Set 3, phosphate-free imipramine hydrochloride titration and solubility data

| vial | *m*_ImpHCl_ / g | *V*_NaCl_ / mL^a^ | pH_initial_ | *V*_NaOH_ /µL^b^ | *V*_HCl_ / µL^c^ | pH_final_ | *S* / mmol L^-1^ | log(*S* / mol L^-1^) |
| --- | --- | --- | --- | --- | --- | --- | --- | --- |
| 1^d^ | 0.05055 | 1.000 | 5.49 | 100.0 | – | 7.37 | 73.4 | -1.13 |
| 2 | 0.05720 | 1.000 | 6.14 | 190.0 | – | 8.11 | 1.85 | -2.71 |
| 3^e^ | 0.05010 | 1.000 | 6.34 | 174.0 | 3.0 | 8.98 | 0.297 | -3.53 |
| 4^e^ | 0.05215 | 1.000 | 6.15 | 225.0 | 37.0 | 10.16 | 0.0367 | -4.44 |
| 5^e^ | 0.05010 | 1.000 | 6.40 | 200.0 | – | 11.87 | 0.0222 | -4.65 |

^a^0.15 mol L^-1^ NaCl; ^b^*c*_NaOH_ = 0.9083 mol L^-1^; ^c^*c*_HCl_ = 0.9725 mol L^-1^; ^d^Solubility above CAC of ImpHCl (CAC=26.2 mmol L^-1^ in 0.15 mol L^-1^ NaCl at 25.0 ± 0.1**°**C. pH=5.92 – 5.17); ^e^Degradation products present in supernatant.

**Table S4.** Set 4, imipramine titration and solubility data

| Vial | pH_initial_ | *V*_HCl_ / µL^a^ | pH_final_ | *S* / mmol L^-1^ | log(*S* / mol L^-1^) |
| --- | --- | --- | --- | --- | --- |
| 1 | 6.33 | 700.0 | 2.67 | 5.35 | -2.27 |
| 2 | 6.24 | 600.0 | 3.46 | 3.52 | -2.45 |
| 3 | 6.29 | 570.0 | 4.13 | 3.56 | -2.45 |
| 4 | 6.18 | 500.0 | 4.85 | 3.77 | -2.42 |
| 5 | 6.30 | 300.0 | 5.40 | 2.02 | -2.69 |
| 6 | 6.25 | 100.0 | 5.95 | 0.897 | -3.05 |
| 7 | 6.28 | – | 6.27 | 0.580 | -3.24 |

^a^ *c*_HCl_ = 1.0729 mol L^-1^

**Table S5.** Set 5, phosphate-free imipramine titration and solubility data

| Vial | pH_initial_ | *V*_HCl_ / µL^a^ | *V*_NaOH_ / µL^b^ | pH_final_ | *S* / mmol L^-1^ | log(*S* / mol L^-1^) |
| --- | --- | --- | --- | --- | --- | --- |
| 1 | 8.00 | 125.0 | 55.0 | 2.62 | 4.89 | -2.31 |
| 2 | 7.98 | 120.0 | 10.0 | 2.94 | 5.85 | -2.23 |
| 3 | 8.07 | 115.0 | 40.0 | 5.30 | 5.78 | -2.24 |
| 4 | 8.16 | 135.0 | 7.0 | 6.27 | 6.31 | -2.20 |
| 5 | 7.91 | 110.0 | – | 7.50 | 3.43 | -2.46 |
| 6 | 7.96 | 135.0 | – | 7.71 | 3.59 | -2.45 |
| 7 | 7.93 | – | – | 8.00 | 1.71 | -2.77 |

^a^ *c*_HCl_ = 1.0729 mol L^-1^; ^b^ *c*_NaOH_ = 1.0621 mol L^-1^

**Table S6.** Set 6, chloride-free imipramine titration and solubility data

| vial | pH_initial_ | *V*_NaOH_ / µL^a^ | pH_final_ | *S* / mmol L^-1^ | log(*S* / mol L^-1^) |
| --- | --- | --- | --- | --- | --- |
| 1 | 2.27 | – | 2.50 | 1.33 | -2.88 |
| 2 | 2.32 | 60.0 | 2.63 | 1.11 | -2.95 |
| 3 | 2.29 | 200.0 | 2.96 | 1.90 | -2.72 |
| 4 | 2.28 | 300.0 | 3.66 | 1.79 | -2.75 |
| 5 | 2.35 | 350.0 | 4.55 | 1.69 | -2.77 |
| 6 | 2.31 | 500.0 | 5.37 | 1.67 | -2.78 |
| 7 | 2.36 | 800.0 | 5.88 | 1.39 | -2.86 |
| 8 | 2.36 | 700.0 | 6.16 | 1.35 | -2.87 |
| 9 | 2.46 | 1000.0 | 6.37 | 1.21 | -2.92 |

^a^ *c*_NaOH_=1.0621 mol L^-1^


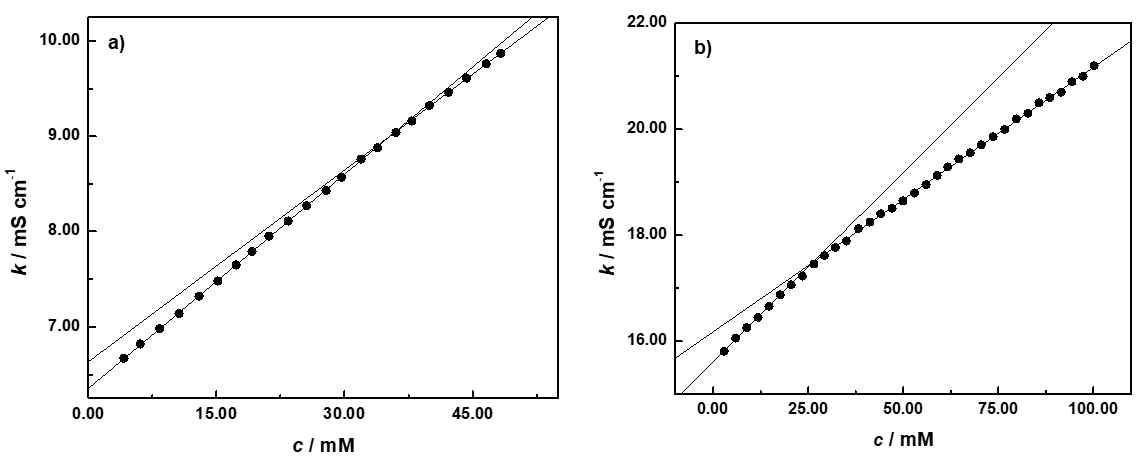


**Figure S1.** Conductometric titration curves of ImpHCl at 25.0 ± 0.1 °C in. (a) 0.10 mol L^-1^ NaH_2_PO_4_ (pH was 4.45 at the beginning of titration and 4.33 in the end of titration) and (b) 0.15 mol L^-1^ NaCl (pH was 5.92 at the beginning of titration and 5.17 in the end of titration)


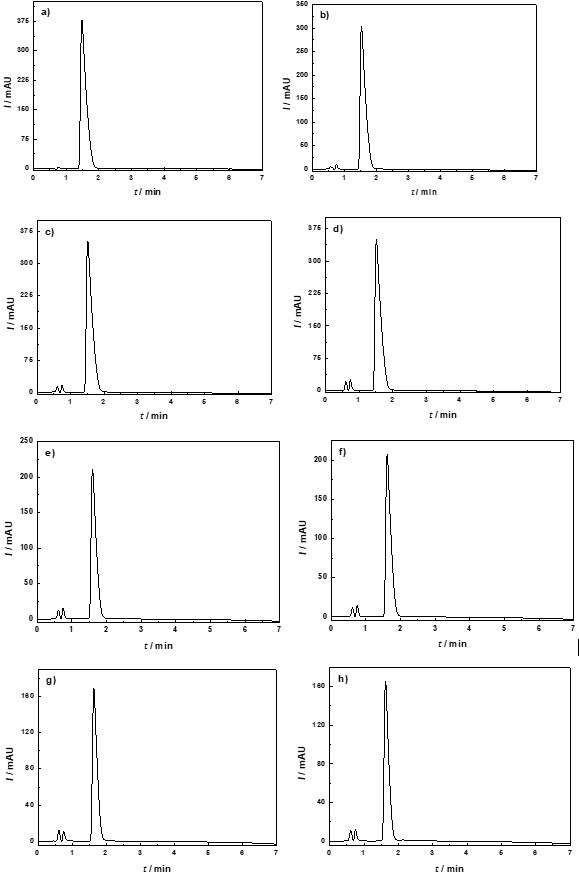


**Figure S2.** HPLC Chromatograms for Set 1 samples 1 to 8 at different pH values: (a) Sample 1 (pH 4.06, diluted 200×), (b) Sample 2 (pH 4.99, diluted 40×), (c) Sample 3 (pH 5.81, diluted 20×), (d) Sample 4 (pH 6.85, diluted 10×),
(e) Sample 5 (pH 8.09, diluted 10×), (f) Sample 6 (pH 8.11, diluted 10×), (g) Sample 7 (pH 8.15, diluted 10×),
(h) Sample 8 (pH 8.17, diluted 10×)

1. **200.0 400.0 600.0**

***c* / μM**

**Figure S3.** Calibration diagram for imipramine (concentration range 9.89 to 592 μmol L^-1^),
linear fit: peak area = 9.6655×10^6^*c* -25.9765; *r*^2^=0.9989


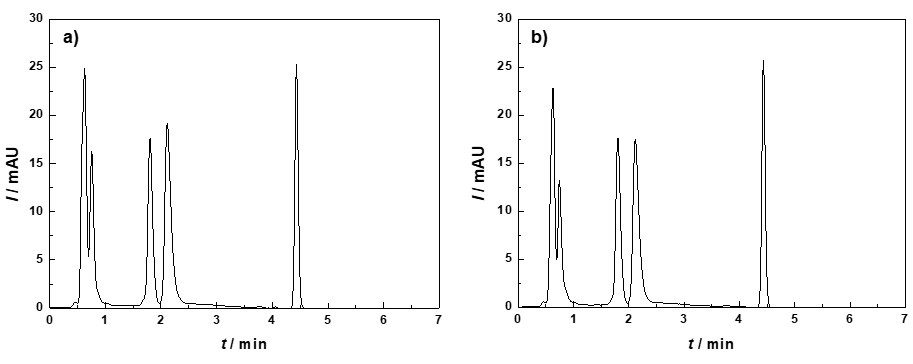


**Figure S4.** HPLC Chromatograms for Set 1 samples at different pH values (degradation products present):
(a) Sample 9 (pH 10.77, diluted 2×), (b) Sample 10 (pH 11.43, diluted 2×). Detection wavelength: 252 nm


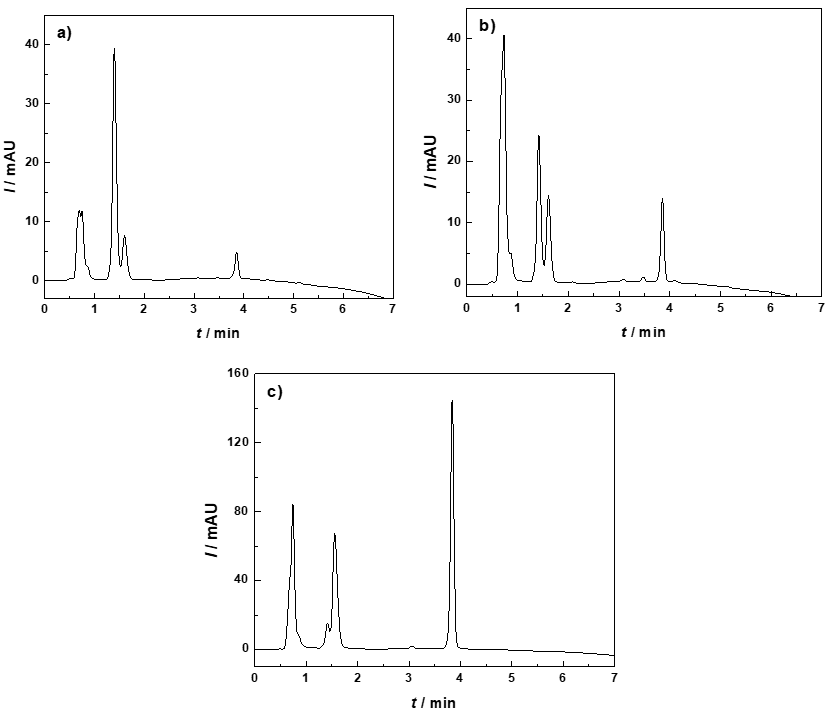


**Figure S5.** HPLC Chromatograms for Set 3 samples at different pH values (degradation products present):
(a) Sample 4 (pH 8.98, diluted 10×), (b) Sample 5 (pH 10.16, diluted 2×), (c) Sample 6 (pH 11.87, diluted 2×).
Detection wavelength: 252 nm
